# Supplementary material for: Causal Relationship between Aging and Anorexia Nervosa: A White-Matter-Microstructure-Mediated Mendelian Randomization Analysis
Source: Biomedicines. 2024 Aug 16;12(8):1874. doi: 10.3390/biomedicines12081874 (PMC11351342; doi:10.3390/biomedicines12081874)
Supplement: Supplementary file 1 [file biomedicines-12-01874-s001.zip › Supplementary Figure/Figure S4/Singe SNP plot of MR analysis for TL to ubm-b-1782.pdf]

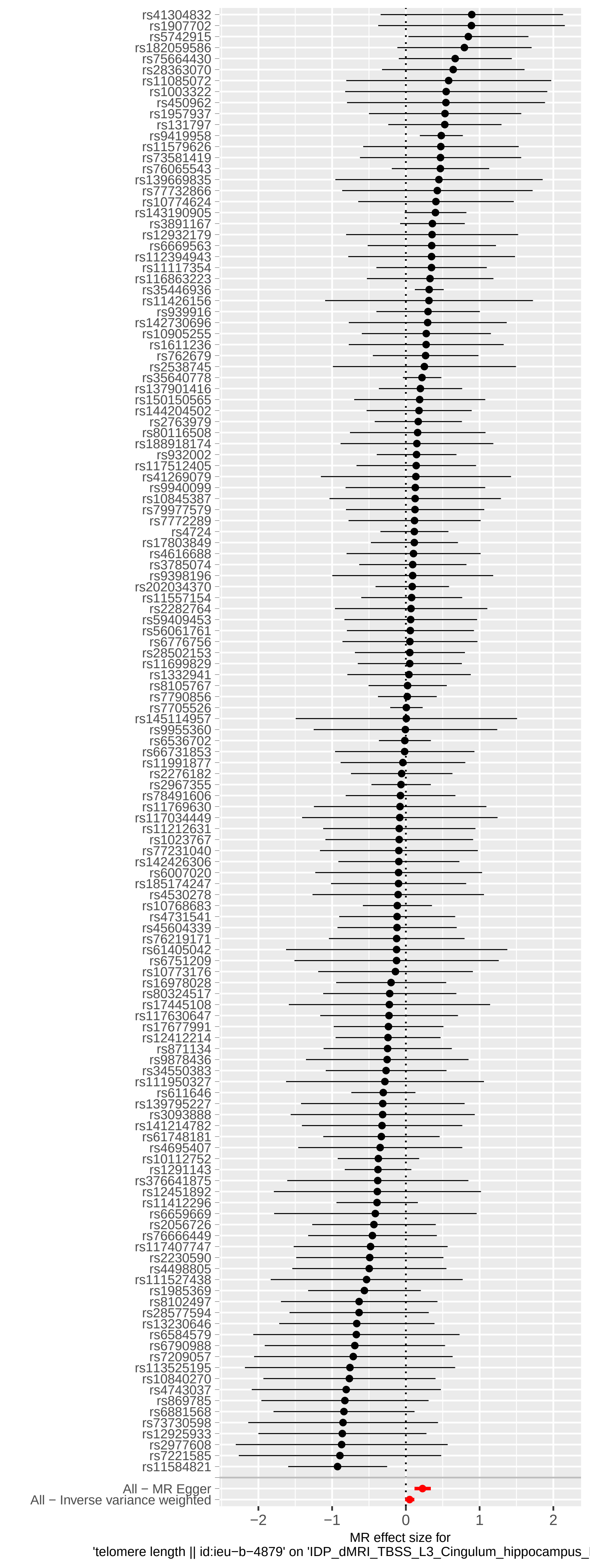

rs41304832
rs1907702
rs5742915
rs182059586
rs75664430
rs28363070
rs11085072
rs1003322
rs450962
rs1957937
rs131797
rs9419958
rs11579626
rs73581419
rs76065543
rs139669835
rs77732866
rs10774624
rs143190905
rs3891167
rs12932179
rs6669563
rs112394943
rs11117354
rs116863223
rs35446936
rs11426156
rs939916
rs142730696
rs10905255
rs1611236
rs762679
rs2538745
rs35640778
rs137901416
rs150150565
rs144204502
rs2763979
rs80116508
rs188918174
rs932002
rs117512405
rs41269079
rs9940099
rs10845387
rs79977579
rs7772289
rs4724
rs17803849
rs4616688
rs3785074
rs9398196
rs202034370
rs11557154
rs2282764
rs59409453
rs56061761
rs6776756
rs28502153
rs11699829
rs1332941
rs8105767
rs7790856
rs7705526
rs145114957
rs9955360
rs6536702
rs66731853
rs11991877
rs2276182
rs2967355
rs78491606
rs11769630
rs117034449
rs11212631
rs1023767
rs77231040
rs142426306
rs6007020
rs185174247
rs4530278
rs10768683
rs4731541
rs45604339
rs76219171
rs61405042
rs6751209
rs10773176
rs16978028
rs80324517
rs17445108
rs117630647
rs17677991
rs12412214
rs871134
rs9878436
rs34550383
rs111950327
rs611646
rs139795227
rs3093888
rs141214782
rs61748181
rs4695407
rs10112752
rs1291143
rs376641875
rs12451892
rs11412296
rs6659669
rs2056726
rs76666449
rs117407747
rs2230590
rs4498805
rs111527438
rs1985369
rs8102497
rs28577594
rs13230646
rs6584579
rs6790988
rs7209057
rs113525195
rs10840270
rs4743037
rs869785
rs6881568
rs73730598
rs12925933
rs2977608
rs7221585
rs11584821
All − MR Egger
All − Inverse variance weighted
−2
−1
0
1
2
MR effect size for
'telomere length || id:ieu−b−4879' on 'IDP_dMRI_TBSS_L3_Cingulum_hippocampus_
